# CLASSIC ERYTHEMA MIGRANS RASH IN PEDIATRIC PATIENT WITH DISSEMINATED LYME DISEASE AND CONCERN FOR LYME MENINGITIS

**DOI:** 10.51894/001c.123047

**Published:** 2024-08-30

**Authors:** Brandon Popp

**Affiliations:** 1 Emergency Medicine Sparrow Hospital

## 65

### INTRODUCTION

Lyme Disease is a tick-borne illness that was first described by the German Physician, Alfred Buchwald, over 130 years ago. However, it was not recognized in the United States until the 1960’s after individuals in Lyme, Connecticut presented with a rash that was quickly followed by arthritis like symptoms after being bit by a tick.

### CASE DESCRIPTION

Patient is a 10-year-old female initially presenting to the emergency department with the chief complaint of fever that has been present for 9 days. On the day of ED presentation, the patient developed a rash on the back of her legs that looked like “circles.” Lyme IgM Western Blot test was positive. She was admitted to general pediatric floor for concerns of suspected meningitis versus early disseminated Lyme disease. She was continued on Vancomycin and Ceftriaxone for meningitis, as well as Lyme disease coverage, and discharged to home with prescription for a 12-day course of oral Doxycycline, after remaining afebrile for 48 hours in the hospital.

### DISCUSSION

Erythema migrans rash develops in 70-80% of diagnosed Lyme disease cases. Disseminated Lyme disease occurs when patients are either not treated, or if Lyme disease was not diagnosed during the initial local stage. 60% of untreated or unrecognized Lyme disease cases progress to disseminated Lyme disease. CSF analysis is not required to make the diagnosis of Lyme meningitis. However lumbar puncture and CSF analysis may assist in ruling out other causes of neurologic symptoms. Historically, parenteral antibiotics have been the treatment of choice for Lyme meningitis. More recently, the efficacy of oral antibiotic therapy, has been explored for Lyme meningitis, and has been deemed to be an equally effective alternative to parenteral antibiotics.

### CONCLUSION

This patient presentation provides an excellent example of the classic erythema migrans rash. Due to the patient’s symptoms of arthralgias and neck pain, the patient was treated for Lyme meningitis.

